# Integrating Chronic Disease Management and Harm Reduction for Youth with Juvenile Idiopathic Arthritis Amid Canada’s Overdose Crisis

**DOI:** 10.3390/children11121424

**Published:** 2024-11-26

**Authors:** Babatope O. Adebiyi, Kathryn A. Birnie, Heinrike Schmeling

**Affiliations:** 1 Section of Rheumatology, Department of Paediatrics, Alberta Children’s Hospital, Cumming School of Medicine, University of Calgary, 28 Oki Drive NW, Calgary, AB T3B 6A8, Canada; heinrike.schmeling@albertahealthservices.ca; 2 Departments of Anesthesiology, Perioperative, and Pain Medicine, Community Health Sciences, Cumming School of Medicine, University of Calgary, Calgary, AB T2N 1N4, Canada; kathryn.birnie@ucalgary.ca; 3 Department of Psychology, Faculty of Arts, University of Calgary, Calgary, AB T2N 1N4, Canada

**Keywords:** overdose crisis Canada, juvenile idiopathic arthritis, opioid use disorder, harm reduction strategy, chronic disease management, multidisciplinary care model, youths, mental health

## Abstract

Juvenile idiopathic arthritis (JIA) is a chronic autoimmune condition in children that often requires long-term pain management, which can include opioid use. In the context of Canada’s ongoing overdose crisis, youth with JIA face risks due to potential opioid dependency and exposure to toxic drug supplies. This commentary proposes an integrated approach combining chronic disease management with harm reduction strategies specifically tailored for JIA patients. By incorporating multidisciplinary care, opioid stewardship, and harm reduction measures, this approach aims to address the dual challenges of managing chronic pain and mitigating substance use risks. Key recommendations include the development of integrated care models, enhanced access to multidisciplinary services, allocation of resources for specialized pain management, research, and mental health support, and investment in harm reduction initiatives. Additionally, comprehensive training for healthcare providers on the intersection of chronic pain, substance use, and mental health is essential. This integrated strategy not only supports the medical and psychosocial needs of youth with JIA but also offers a model for addressing the broader challenges faced by vulnerable populations in the overdose crisis. Adopting these measures will help protect this at-risk group, improve their quality of life, and contribute to the overall public health response to the overdose epidemic.

## 1. Introduction

Juvenile idiopathic arthritis (JIA) is the most common chronic inflammatory rheumatic disease in childhood, with an estimated incidence of 10–20 per 100,000 children below the age of 16 years. According to the International League Against Rheumatism (ILAR) classification, patients can be classified into seven subgroups depending on the number of affected joints during the onset and course of the disease and the presence of extraarticular manifestations [1]. Symptoms typically intensify in the morning or following inactivity. Beyond joint involvement, some children may present with systemic manifestations, including fever, lymphadenopathy, and rashes. JIA may also affect the eyes (e.g., uveitis) and skin (e.g., psoriasis), resulting in additional complications [2]. The management of JIA involves a multidisciplinary approach, including medication, physical therapy, and psychosocial support, to control symptoms and improve quality of life [3].

In Canada, the toxic drug overdose crisis has emerged as a significant public health challenge, disproportionately affecting vulnerable populations, including youth with chronic conditions. The crisis is characterized by the widespread availability of toxic drug supplies, leading to increased rates of substance use and overdose deaths [4]. Youth with JIA may be particularly at risk due to the overlapping burdens of chronic pain, mental health, and potential exposure to opioids for pain management [5]. This exposure, combined with the psychosocial stressors of living with a chronic illness, can increase the risk of developing substance use disorders [2]. Given the intersection of pain management needs and the opioid epidemic, this commentary explores integrated care strategies to protect these vulnerable youths.

The objective of this commentary is to propose an integrated approach that combines chronic disease management with harm reduction strategies for JIA patients at risk of substance use.

## 2. Burden of JIA and the Role of Pain Management

Managing chronic pain in youth with JIA presents significant challenges due to the multifactorial nature of pain, which affects physical, emotional, social, and school functioning [6]. Despite advances in treatment, many children with JIA continue to experience clinically significant pain, which negatively impacts their quality of life [7]. The biopsychosocial model of pain management is recommended, emphasizing a multidisciplinary approach that includes physicians, nurses, physiotherapists, occupational therapists, and psychologists [7].

The standard pharmacological treatments for JIA include nonsteroidal anti-inflammatory drugs, disease-modifying antirheumatic drugs, and biologics to control inflammation and prevent joint damage [8]. A multimodal approach that integrates pharmacological, psychosocial, and physical strategies is critical to quality pediatric pain management [9]. For moderate to severe persistent pain, opioids may be considered, although they are not the first line of treatment due to potential risks [6]. However, for some youths with JIA, their long-term use becomes unavoidable due to inadequate relief from other treatments, posing an elevated risk for opioid dependency. Physical therapies are crucial for maintaining joint function and muscle strength [7]. Psychological interventions, including cognitive–behavioral therapy, biofeedback, relaxation techniques, and mindfulness-based stress reduction (MBSR), are also key components of pain management, helping patients develop effective coping strategies [6].

## 3. Canada’s Overdose Crisis: Impact on Youths

The opioid crisis in Canada has become a significant public health emergency, with a staggering increase in opioid-related deaths and hospitalizations over recent years. Between January 2016 and December 2023, 44,592 apparent opioid toxicity deaths were reported in Canada [10]. The crisis is driven by both illegal and prescription opioids, with fentanyl and its analogs being major contributors to the rising death toll [11]. The COVID-19 pandemic has further exacerbated the situation, leading to higher rates of fatal overdoses [12].

Youths, particularly those with chronic conditions such as JIA, are uniquely vulnerable to this crisis. These individuals often encounter opioids medically, for pain management, or for non-medical use [13]. This exposure increases the potential for dependency and opioid use disorders, especially when combined with the psychosocial stressors of living with a chronic illness. The dual burden of chronic pain and the risk of substance use disorders presents a significant challenge for youth with JIA. Chronic pain can lead to increased opioid use, which, in turn, increases the risk of developing an opioid use disorder [14]. The contaminated illegal drug supply, particularly with fentanyl, further increases the risk of accidental overdose [11]. Youths managing chronic pain are at risk of opioid dependency, which underscores the need for integrated care approaches that combine effective multimodal pain management with harm reduction strategies [14]. Recent national standards for pediatric pain management offer guidance for how opioid prescribing and use for pain can be performed safely, effectively, and equitably. Recommendations include ensuring multimodal pain management, using the lowest effective dose, assessing long-term opioid use risk, incorporating psychosocial supports to address higher-risk use, and working with families to develop individualized pain care plans (i.e., plans for safe discontinuation, storage, disposal, and tapering) [9].

## 4. Integrating Chronic Disease Management and Harm Reduction

The integration of chronic disease management with harm reduction strategies for youth with JIA is a comprehensive strategy that addresses both medical and psychosocial needs, addressing the dual challenges of managing chronic pain and mitigating opioid-related risks [15].

The incorporation of harm reduction programs within chronic pain clinics in British Columbia has demonstrated a decrease in opioid-related harm while ensuring appropriate pain management for patients with chronic diseases [16]. This example integrates harm reduction initiatives, including opioid stewardship, safe prescribing practices, and naloxone accessibility, within chronic disease management frameworks. In the future, anti-fentanyl monoclonal antibodies can be accessible once commercially available. These antibodies offer several advantages, including prolonged protection against fentanyl and its analogs, the potential for prophylactic use in high-risk individuals, and broad neutralization capabilities that can counteract a wide range of fentanyl analogs [17]. These features make them a promising alternative to current treatments like naloxone. This model underscores the potential benefit of using a similar procedure for youth with JIA, in which opioids may be utilized to alleviate chronic pain.

A multidisciplinary care model is essential as a collaborative approach involving various healthcare professionals. Rheumatologists focus on managing the autoimmune aspects of JIA with medications such as Nonsteroidal Anti-Inflammatory Drugs (NSAIDs), steroids, Disease-Modifying Antirheumatic Drugs (DMARDs), and biologics while monitoring for disease progression and treatment side effects [18]. Medications used to reduce pain or control inflammation in children with JIA may suppress the immune system, making them more susceptible to infections. However, regular monitoring, vaccination, hygiene practices, early disease control and prompt treatment, patient education, and medication adjustments can effectively reduce the number of infections while maintaining the control of JIA symptoms. Multidisciplinary pain specialists codevelop individualized pain management plans that include pharmacological, physical, and psychosocial interventions, which have evidence of reduced pain intensity in youth with chronic arthritis [19,20,21]. Addiction counselors and/or psychologists can provide support for youth at risk of substance use, helping them build resilience and develop coping strategies [6]. Mental health professionals play a critical role in addressing psychological burdens, such as anxiety and depression, which are common among youth with JIA [22]. They can use behavioral therapy (a type of psychotherapy that focuses on changing maladaptive behaviors through various techniques and interventions) such as cognitive behavioral therapy, exposure therapy, systematic desensitization, reinforcement strategies, biofeedback, and MBSR to manage these burdens.

Harm reduction measures are vital in this integrated approach. Canada’s national health standard for pediatric pain management directs healthcare teams to identify and address risk factors associated with higher-risk opioid use and/or opioid use disorder and provide individualized pain care plans that include education about opioids [9]. The standard also indicates that a child with risk factors for higher-risk use or opioid use disorder should receive additional support, including coordinated care, adjusted prescribing schedules to optimize benefits and minimize harm, co-prescription of naloxone, and documentation of individualized care plans for communication, care transitions, and evaluation of the impact of pain management [9]. Opioid stewardship programs (careful management of opioid use) promote the careful use of opioids, ensuring that they are prescribed only when necessary [23]. Education on safe opioid use is crucial, providing youth and their families with information about the risks and safe use of opioids emphasizing adherence to prescribed dosages [24]. Ensuring access to naloxone, an opioid overdose reversal drug, is essential, and training on its use can empower youth and their families to respond effectively in the event of an overdose [25].

**Figure 1:**
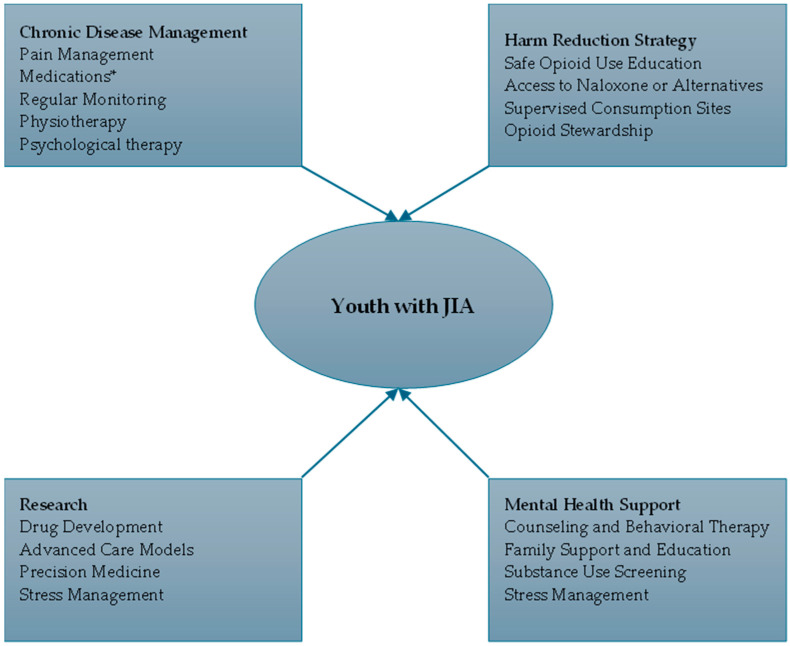
Enhanced integrated care model for youths with JIA amid Canada’s overdosing crisis. * Medications: Nonsteroidal Anti-Inflammatory Drugs (NSAIDs) {e.g., Ibuprofen, Naproxen}; Disease-Modifying Antirheumatic Drugs (DMARDs) {e.g., Methotrexate, Sulfasalazine, Leflunomide}; Biologics {e.g., Tumor Necrosis Factor (TNF) Inhibitors, Interleukin-1 (IL-1) Inhibitors, Interleukin-6 (IL-6) Inhibitors}; Corticosteroids {e.g., Prednisone}, and Intra-Articular Steroids {e.g., Triamcinolone acetonide}.

Harm reduction strategies, such as ensuring access to naloxone and supervised consumption sites, should be integrated into the overall care plan for youths with JIA receiving opioids. When combined with robust chronic pain management, these measures not only mitigate overdose risks but also improve overall treatment adherence and patient outcomes. Figure 1 shows the enhanced integrated care model for youths with JIA amid Canada’s overdosing crisis

This proposed approach methods for managing JIA amid Canada’s overdose crisis may face numerous challenges, encompassing the intricacy of coordinating multidisciplinary care, the necessity for sufficient funding and resources, extensive training for healthcare practitioners, restricted access to services in rural regions, and the stigma linked to opioid utilization and mental health concerns. Alternatives to tackle these obstacles comprise expanding telemedicine services to enhance access to specialized care, formulating community-based initiatives that capitalize on local resources, advocating for policies that prioritize financing for integrated care and harm reduction, implementing public education campaigns to reduce stigma, promoting education programs for youth with JIA, and establishing peer support networks to provide emotional and social support for youth managing JIA and substance use risks. These strategies can contribute to amplifying the efficacy of integrated care methodologies and improving outcomes for youth with JIA.

## 5. Recommendations for Policy and Practice

Enhancing access to multidisciplinary services: Policies should ensure that youth with JIA have access to comprehensive, multidisciplinary healthcare services. This includes removing barriers to accessing mental health services, addiction counseling, and multidisciplinary specialized pain management programs [19]. Policymakers should invest in telemedicine services and mobile pain management units to reach youth in rural or underserved areas, where access to specialized care is often limited. They can provide financial incentives for health providers to participate in telemedicine programs. Also, policymakers should expand insurance coverage for telemedicine services and streamline referral processes to reduce wait times and improve coordination.

Support integrated care models: When prescribing opioids, policymakers should support the development of integrated care models for youth with JIA, combining chronic disease management with harm reduction strategies. The government should hire a care coordinator to facilitate communication among rheumatologists, multidisciplinary pain specialists, addiction counselors, and mental health professionals, thereby offering a comprehensive and evidence-based multimodal treatment approach [26].

Allocate resources for chronic pain management: Governments and healthcare organizations should allocate funding for specialized programs that provide multimodal chronic pain management for youth with JIA, as is recommended in Canada’s national health standard for pediatric pain [9]. These programs should incorporate pharmacological, psychosocial, and physical interventions [23]. Annual audits should track how effectively these resources are utilized.

Invest in harm reduction initiatives: Policymakers should expand access to harm reduction initiatives that support youth at risk of substance use. This includes expanding access to naloxone kits, supervised consumption sites, and educational programs on safe medication use [27].

Develop comprehensive training programs: Healthcare providers should receive comprehensive training on chronic disease management and harm reduction, covering best practices and core competencies for managing chronic pain in youth with JIA, safe opioid prescribing, and substance use disorder identification [9]. Training should be made accessible through online platforms, with incentives such as certification and continuous professional development credits to encourage participation.

Evaluating long-term outcomes of integrated care models: The long-term success of these integrated care models should be evaluated using specific metrics. Key indicators of success would include a reduction in opioid prescriptions, improved pain management outcomes, fewer opioid-related overdoses or substance use-related incidents, and enhanced overall quality of life for JIA patients. Ongoing monitoring of these outcomes can ensure that integrated care approaches remain effective in balancing chronic pain management and harm reduction strategies. Regular data collection, patient feedback, and public health evaluations will be critical in assessing the long-term impact of these interventions on both individual and public health levels.

## 6. Call to Action

With opioid overdose deaths at unprecedented levels, immediate action is needed to protect youth with JIA from falling through the cracks. An urgent call for integrated care, policy innovation, and harm reduction strategies will not only safeguard the health of these vulnerable youth but also provide a model for managing chronic disease amid public health crises.

## 7. Conclusions

The integration of chronic disease management, mental health supports, chronic pain management, research, and harm reduction strategies are crucial for youth with JIA in Canada, especially during the overdose crisis. This holistic approach combines medical, psychological, and social support to manage the condition effectively while minimizing opioid dependency and opioid use disorder. While the proposed model emphasizes the importance of multidisciplinary care, further steps are essential to ensure its success. Future research should focus on developing new, safer, and more effective medications (such as anti-fentanyl monoclonal antibodies), targeted non-opioid therapies, and exploring innovative care models. Healthcare providers should adopt multidisciplinary approaches, policymakers should prioritize funding and policy initiatives that support integrated care frameworks (including funding for telemedicine and mobile health services to reach underserved communities), and advocacy groups should champion integrated care approaches to protect vulnerable youth. By advancing research, improving access to integrated care, and ensuring healthcare providers receive comprehensive training, we can better protect JIA patients from opioid dependency and improve their overall quality of life. This comprehensive approach not only addresses the immediate needs of youth with JIA but also offers a model for managing other chronic conditions amid public health crises.

## Data Availability

No new data were created or analyzed in this study. Data sharing is not applicable to this article.

## Abbreviations

JIA: Juvenile Idiopathic Arthritis, ILAR: International League Against Rheumatism, MBSR: Mindfulness-Based Stress Reduction, NSAIDs: Nonsteroidal Anti-Inflammatory Drugs, DMARDs: Disease-Modifying Antirheumatic Drugs, TNF: Tumor Necrosis Factor, COVID: Coronavirus Disease, IL: Interleukin.
